# Computer-assisted planning vs. conventional surgery for the correction of symptomatic mid-shaft clavicular nonunion and malunion

**DOI:** 10.1016/j.jseint.2023.07.005

**Published:** 2023-07-28

**Authors:** Bettina Hochreiter, Laura Victoria Saager, Christoph Zindel, Anna-Katharina Calek, Christoph Stern, Karl Wieser, Christian Gerber

**Affiliations:** a Department of Orthopedics, Balgrist University Hospital, University of Zurich, Zurich, Switzerland; b Department of Radiology, Balgrist University Hospital, University of Zurich, Zurich, Switzerland; c Balgrist Campus, Orthopaedic Research Center, Zürich, Switzerland

**Keywords:** Clavicle, Nonunion, Malunion, Patient specific instrumentation, PSI, ORIF

## Abstract

**Background:**

The aim of this study was to compare the clinical and radiographic outcomes of treatment of symptomatic mal- and/or nonunion of midshaft clavicle fractures using radiographically based free-hand open reduction and internal fixation (ORIF) or computer-assisted 3D-planned, personalized corrective osteotomies performed using patient-specific instrumentation (PSI) and ORIF. The hypotheses were that (1) patients treated with computer-assisted planning and PSI would have a better clinical outcome, and (2) computer-assisted surgical planning would achieve a more accurate restoration of anatomy compared to the free-hand technique.

**Methods:**

Between 1998 and 2020, 13 patients underwent PSI, and 34 patients underwent free-hand ORIF and/or corrective osteotomy. After application of exclusion criteria, 12/13 and 11/34 patients were included in the study. The clinical examination included measurement of the active range of motion and assessment of the absolute and relative Constant–Murley Scores and the subjective shoulder value. Subjective satisfaction with the cosmetic result was assessed on a Likert scale from 0 to 100 (subjective aesthetic value). 11/13 and 6/11 patients underwent postoperative computed tomography evaluation of both clavicles. Computed tomography scans were segmented to generate 3D surface models. After projection onto the mirrored contralateral side, displacement analysis was performed. Finally, bony union was documented. The average follow-up time was 43 months in the PSI and 50 months in the free-hand cohort.

**Results:**

The clinical outcomes of both groups did not differ significantly. Median subjective shoulder value was 97.5% (70; 100) in the PSI group vs. 90% (0; 100) in the free-hand group; subjective aesthetic value was 86.4% (±10.7) vs. 75% (±18.7); aCS was 82.3 (±10.3) points vs. 74.9 (±26) points; and rCS was 86.7 (±11.3) points vs. 81.9 (±28.1) points. In the free-hand group, 2/11 patients had a postoperative neurological complication. In the PSI cohort, the 3D angle deviation was significantly smaller (PSI/planned vs. free-hand/contralateral: 10.8° (3.1; 23.8) vs. 17.4° (11.6; 42.4); *P* = .020)). There was also a trend toward a smaller 3D shift, which was not statistically significant (PSI/planned vs. free-hand/contralateral: 6 mm (3.4; 18.3) vs. 9.3 mm (5.1; 18.1); *P* = .342). There were no other significant differences. A bony union was achieved in all cases.

**Conclusion:**

Surgical treatment of nonunion and malunions of the clavicle was associated with very good clinical results and a 100% union rate. This study, albeit in a relatively small cohort with a follow-up of 4 years, could not document any clinically relevant advantage of 3D planning and personalized operative templating over conventional radiographic planning and free-hand surgical fixation performed by experienced surgeons.

Clavicle fractures are common injuries, accounting for 5%-10% of all fractures in adults, with an annual incidence of 36.5 per 100,000.^15^ The nonunion rate of dislocated midshaft clavicle fractures is reported to be 14.5% after conservative and 1.4% after surgical treatment,^12^ with symptomatic malunions occurring in 9% of conservatively treated midshaft fractures.^4^ The main goal of surgery in patients with symptomatic clavicle nonunion or malunion is to restore length and alignment in a position that allows anatomical bone healing,^19^ does not compromise brachial plexus or arm function, and restores aesthetics. Posttraumatic malunion of the middle third of the clavicle represents a complex three-dimensional deformity typically consisting of shortening and translation of the clavicle and rotation of the lateral fragment.^13^

Surgical management of symptomatic non- or malunions remains challenging and is associated with perioperative risks such as infection, vascular or nerve damage. Two-thirds of complications are of technical origin and avoidable.^9^^,^^21^ To minimize surgical complications and achieve satisfactory results, accurate and careful surgical planning is recommended for open reduction and internal fixation (ORIF) with or without corrective osteotomy (OT) of the clavicle.^9^ Preoperative planning based on radiographs and computed tomography (CT), however, may not provide sufficient information to quantitatively correct the complex three-dimensional (3D) deformity.^16^ To improve the assessment of the deformity and the accuracy of ORIF with or without OT,^23^ computer-assisted preoperative 3D planning based on the contralateral anatomy has been introduced. With the exact 3D deformity identified, custom-made, personalized cutting and reduction templates should not only allow to obtain healing but also quantitative anatomical reconstruction.

The aim of this study was to compare the clinical and radiographic outcome of treatment of symptomatic mal- and/or nonunion of midshaft clavicle fractures using radiographically based free-hand ORIF or computer-assisted 3D planned, with personalized corrective osteotomies performed using patient-specific instrumentation (PSI) and ORIF. The hypotheses were that (1) patients treated with computer-assisted planning and PSI would have a better clinical outcome, and (2) computer-assisted surgical planning would achieve a more accurate restoration of anatomy compared to the free-hand technique.

## Material and methods

### Study design and patient selection

**Figure 1:**
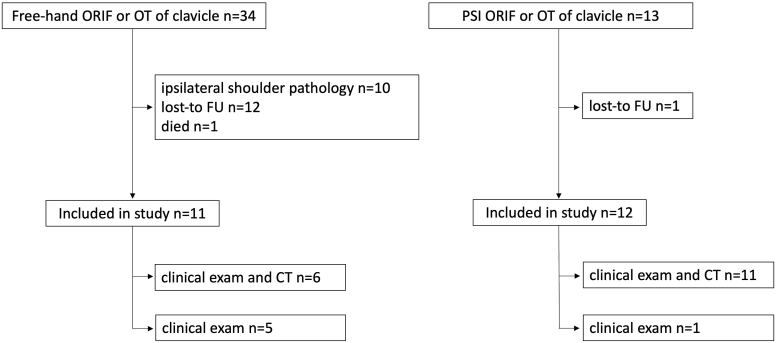
Flow-chart of patient selection for the two cohorts. *CT*, computed tomography; *PSI*, patient-specific instrumentation; *ORIF*, open reduction internal fixation; *OT*, osteotomy; *FU*, follow-up.

The responsible review board approved this comparative cohort study. Medical records and radiographs of all patients presented in our outpatient clinic with a symptomatic non or malunited midshaft clavicle fracture who underwent corrective OTs between 1998 and 2020 were retrospectively reviewed. Symptomatic was defined as pain and/or loss of function. Since 2015, from the time PSI became available at our institution, there were 13 patients who underwent PSI ORIF and/or corrective OT. Before 2015, 34 patients who underwent free-hand ORIF and/or corrective OT were identified. Only two patients underwent conventional surgery after 2015 because the guides were not available at the time of the surgery. Patients who did not have a complete radiographic and clinical follow-up were contacted by telephone and prospectively followed-up. To be included, patients had to have undergone ORIF and/or corrective OT at the authors’ institution and a complete follow-up exam no less than 1 year postoperatively. Patients with shoulder pathology and/or previous surgery of the affected upper extremity (other than the clavicle), which could potentially confound the clinical results, were excluded. Patients in the free-hand group who had a fracture of the contralateral clavicle were also excluded (Fig. 1).

Demographic parameters (age, sex, body mass index, nicotine abuse, and handedness) were assessed. Finally, twelve patients who were treated with PSI ORIF and/or corrective OT and eleven patients treated with free-hand ORIF and/or corrective OT were included. The reason for the difference in the follow-up rate is that with the free-hand technique, the follow-up was completed before the annual follow-up if the outcome was positive. In the case of the 12 patients lost to follow-up, the surgery took place before 2005 and the patients could not be reached by telephone (relocation, change of telephone number). As the PSI technique is a new technique, the patients were routinely seen 1 year after the operation. Of all 47 cases operated on at our institution since 1998, 40 cases were operated on by four fellowship-trained shoulder surgeons who were head or deputy head of the shoulder department at the time of the surgery. Of the final 23 patients included 21 were operated on by 1 of these four shoulder surgeons.

### Preoperative planning for PSI ORIF and/or corrective OT

**Figure 2:**
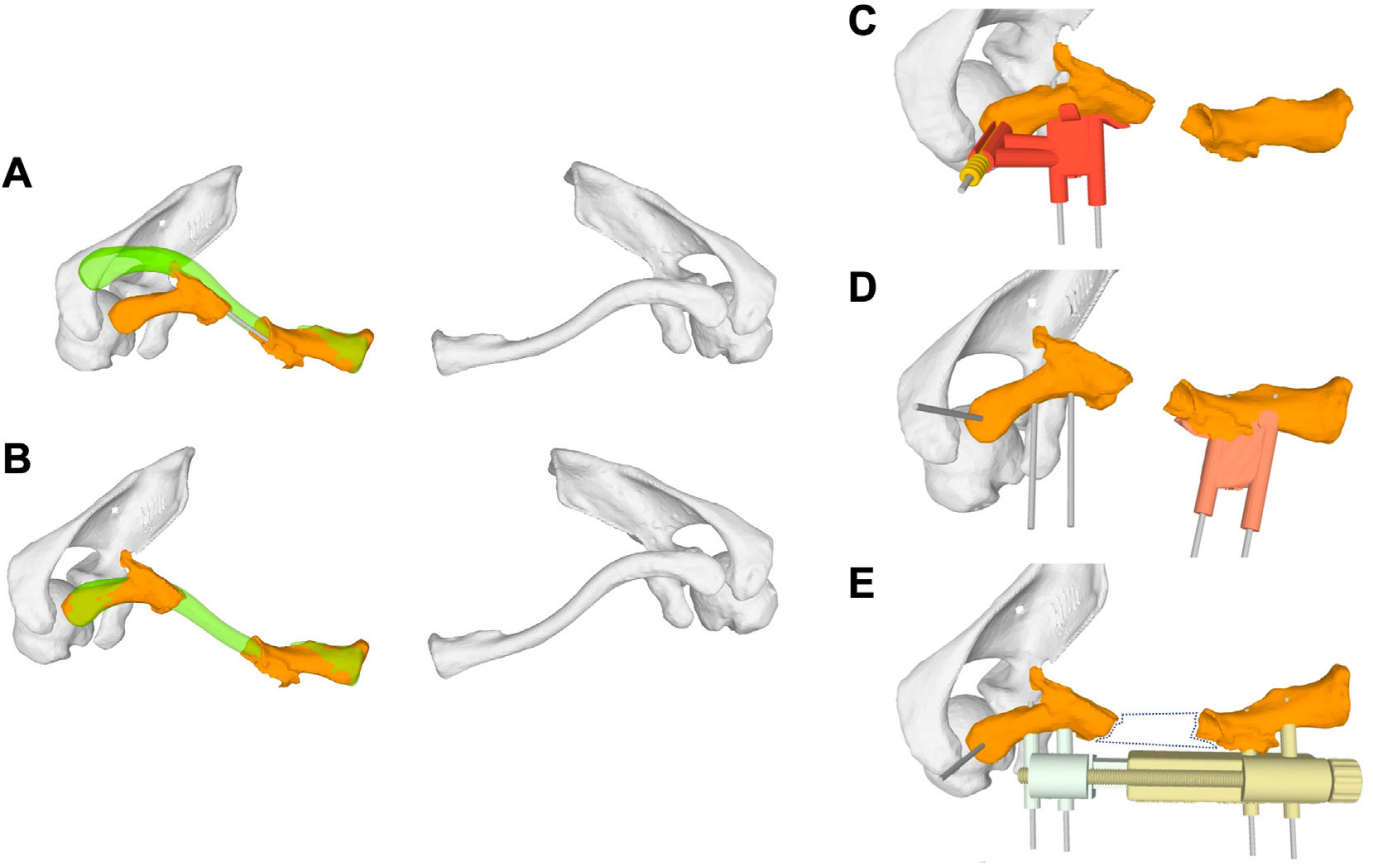
Shows a planning example of a case with nonunion and significant bone loss using the CASPA planning software. (**A**) The medial third of the pathological clavicle (orange) is aligned with the mirrored contralateral clavicle (green). (**B**) The lateral third of the pathological clavicle (orange) along with the scapula is then aligned with the mirrored contralateral clavicle (green) to visualize the desired correction. (**C** and **D**) PSI guides for K-wire placement are planned for the lateral and medial fragment. (**E**) K-wires are used as reduction tools for the reduction guide. The reduction guide has a mark for the planned clavicle length. It has a wheel that can be used to either bring the two ends to length or to compress them after the iliac bone graft has been inserted. The volume of the defect is measured and an appropriate guide for the bone graft is created and 3D-printed. *CASPA*, Computer Assisted Surgery Planning Application.

**Figure 3:**
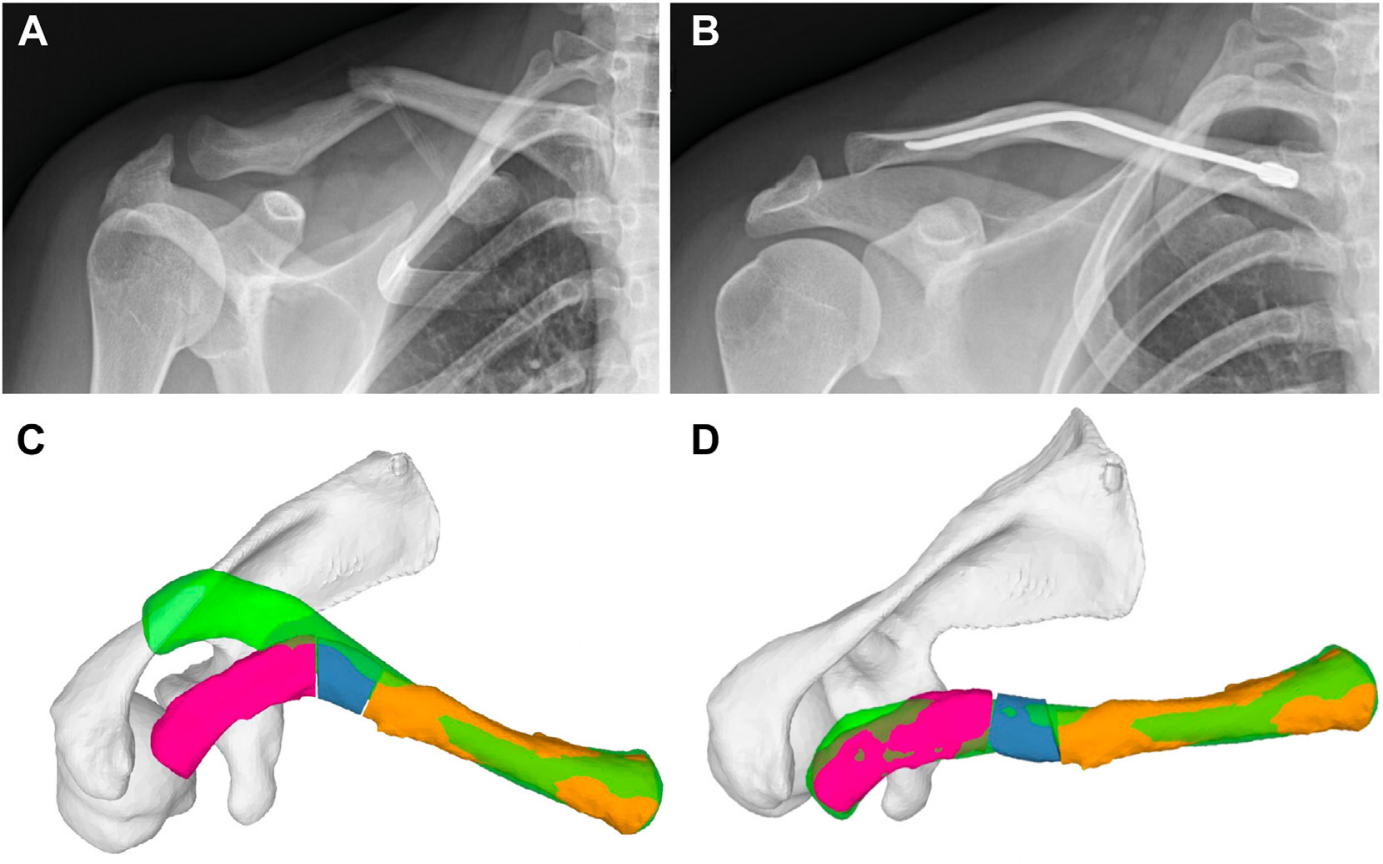
This is a case of a 35-year-old patient with a malunited mid-shaft clavicle fracture after ORIF with a nail (**A** and **B**). The reconstructed 3D models of the patient’s segmented CT data of both clavicles were imported into planning software. The model of the contralateral anatomy (clavicle and scapula) was mirrored and used as a reconstruction template (**C** and **D**).

The basic principle of the computer-assisted planning of posttraumatic clavicle deformities has been described previously.^18^^,^^23^ Therefore, briefly, the patients’ CT data (Siemens Somotom Edge Plus; Siemens; München, Germany, 1 mm slices) were imported into the MIMICS software (Materialise, Leuven, Belgium), and semiautomatic 3D segmentation of the involved and the contralateral clavicles was performed. In order to take into account the relationship to the surrounding bony anatomy, not only the clavicles of both sides but also the scapulae, first ribs, and sternum were extracted. The reconstructed 3D models were then imported into the planning software CASPA (Computer Assisted Surgery Planning Application version 5.0; in-house development Balgrist CARD AG, Zürich, Switzerland). As is the case with other long bones,^16^^,^^22^^,^^24^ the contralateral anatomy is proposed as a reliable reconstruction template for the correction of post-traumatic clavicle deformities. In the preoperative planning software, the models of the contralateral anatomy (clavicle and scapula) were mirrored and used as a reconstruction template (Figs. 2 and 3).

Of the twelve patients included, eight had a nonunion, two had a refracture of a malunion, and two had a malunion.

*Ten patients underwent ORIF*: the nonunion was reduced in the planning software to match the anatomy of the contralateral side as closely as possible. The shape of the contact area or of the resulting gap between the fragments could be modified by adjusting the orientation and position of the planned reduction. An example of a planning procedure for a case with nonunion and significant bone loss is shown in Figure 2.

**Figure 4:**
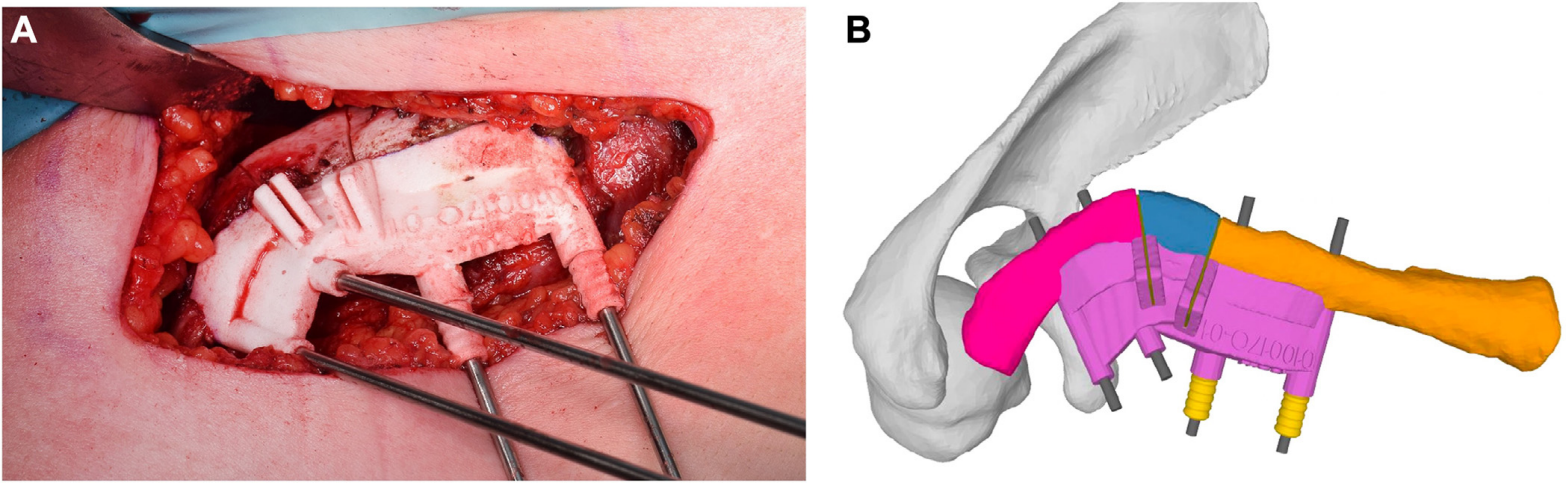
In the case of the 35-year-old patient with malunion, a corrective osteotomy was planned. Figures **A** and **B** show the “basic” cutting guide held in place by two parallel 2.5 mm K-wires on each side. The same K-wires will be used to apply the reduction guide in the next step.

*In the two patients with malunion, a corrective OT was planned* (Fig. 4): the medial and lateral thirds of the affected clavicle were superimposed on the native clavicle. The relative 3D rotational translation difference between the lateral and the medial clavicle segments quantified the deformity. OT and optimal reduction were simulated. Again, the shape of the contact area or of the resulting gap between the fragments could be modified by adjusting the orientation and position of the planned fixation.

While the amount of acceptable shortening has not been clarified clinically, a cadaveric study showed that a 10% shortening of the clavicle affected scapular kinematics.^11^ We defined pathologic clavicle shortening as relevant if the length difference was either larger than 10% or if the resultant contact area seemed to be too small to reliably allow bony healing. In the four out of twelve cases fulfilling these criteria, an iliac crest bone graft was harvested and used as an interpositional, structural autograft. A cutting guide was designed and 3D printed for the exact dimensions of the block.

**Figure 5:**
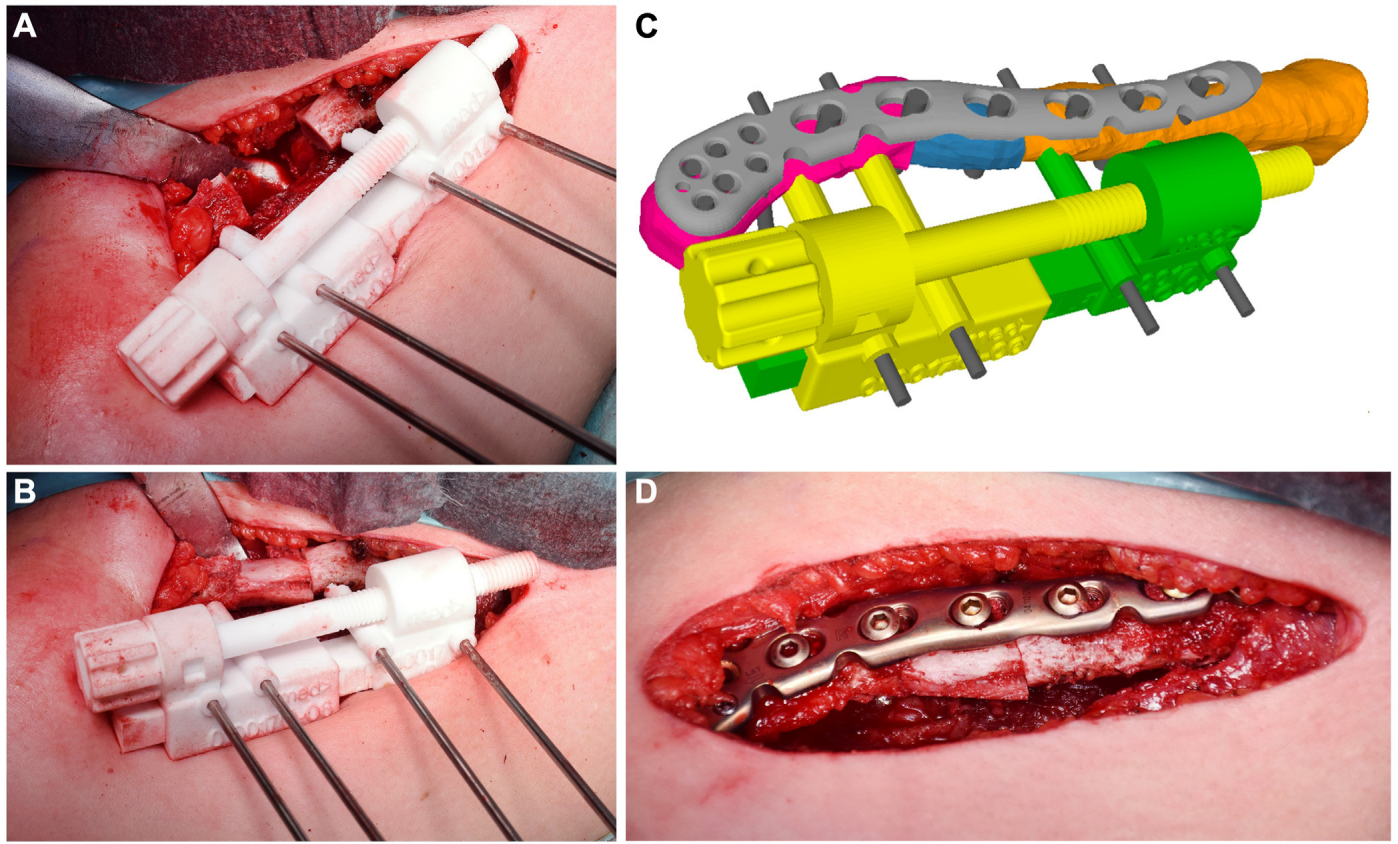
The “basic” guide shown in Figure 3 was removed and the reduction guide, which allows optional interfragmentary compression via a rotatory knob (yellow)—was then positioned over the 2.5 mm K-wires (**A**-**C**). The previously resected bone block (Fig. 3*A*) was used to fill the defect. In this case, the plate was positioned on the superior surface of the clavicle, independent from the reduction guide and the K-wires (**C**-**D**). The holes were filled with screws after drilling and length measurement.

After the simulated correction, the most appropriate surgical implant and its position were determined. For each patient, the surgeon selected an implant that was properly aligned with the characteristics of the fracture;this included either precontoured plates (Fig. 5) that could be aligned with the mirror-model of the contralateral clavicle in the planning software, or implants that were prebent on the 3-D printout of the mirror-model before surgery. Once the planned correction and plate alignment were defined, at least two parallel 2.5-mm K-wires were created in the planning software for each fragment and positioned through the plate holes (Fig. 5). K-wires were used as reference K-wires for the OT guides and/or as reduction K-wires for the reduction guide. A “basic” guide was then designed to conform to the cranial surface of the clavicle, allowing the K-wires to be placed in the medial and lateral parts of the pathological clavicle prior to reduction or OT. This “basic” guide also allowed OT of the malunited clavicle or planned removal of hypertrophic bone. The reduction guide was used to reduce the K-wires and thus both fragments to the planned position. To allow compression plating, K-wires were placed in an eccentric position through the dynamic compression screw holes of the plate in at least 1 of the two fragments.

### Surgical technique

In all cases, an oblique incision was made just anteroinferior to the clavicle, centered over the site of the mal- or nonunion. The platysma was incised transversely. The supraclavicular nerves were cut and coagulated, if previously identified. The clavipectoral fascia was incised to expose the underlying clavicle.

#### PSI ORIF and/or OT

As described above, the planned guides were 3D-printed and sterilized. After intraoperative visualization of the clavicle, the fracture ends were cleaned, and important bony landmarks were prepared. The "basic" guides were then applied and fixed with 2.5 mm K-wires (Fig. 4). In cases where ORIF alone was planned, the hypertrophic callus was resected depending on the situation and preoperative planning. In cases where a corrective OT was planned, an OT was performed using a cutting guide. An iliac crest bone block was used in 4/12 cases, bone from the resected hypertrophic callus was used in 2/12, cases and cancellous bone from the iliac crest was used in 1/12 cases. The reduction guide was then positioned over the 2.5 mm K-wires (Fig. 5). The plate was positioned either beneath the reduction guide if the guide was applied from the top or on the superior surface of the clavicle if the guide was applied from the front. The free holes were filled with screws after drilling and length measurement. At the end of surgery, the K-wires were removed and replaced with screws. After radiological control of the plate and screw position, the site was thoroughly irrigated, the fascia was closed, and then the skin.

#### Free-hand ORIF and/or OT

In the free-hand cohort, ten patients had a nonunion, and 1 patient had a refracture of a malunion. In all cases, the nonunion was thoroughly débrided and freshened until bleeding occurred. Care was taken to preserve soft tissue attachments to the bone fragments. The fracture was then reduced so that at least 50% of the bone ends were in contact. In cases where bone contact was less than 50% or where establishing bone contact would have resulted in unacceptable clavicle shortening, an iliac crest bone block was harvested and placed (2/11). In cases with 50% or more bone contact, cancellous bone (from the clavicle, tibia, or iliac crest) was used in 4/11 cases, and InductOs (Medtronic, Minneapolis, MN, USA) in 1/11 cases. Reduction was then performed, a 3.5 mm prebent locking compression plate clavicle plate was applied, and bridge plating was performed. After radiological control of the plate and screw position, the site was thoroughly irrigated, the fascia closed and then the skin.

Postoperatively, the shoulder was immobilized in a sling for six weeks. Depending on the surgeon's discretion and intraoperative stability, a range of motion up to 90° of active-assistive elevation and abduction was allowed during the first six weeks.

### Clinical assessment

Preoperative and postoperative clinical assessments were performed by an independent investigator who had not operated on the patients. This was done in an institutionally standardized manner. The clinical examination included measurement of the active ROM using a hand-held goniometer and assessment of the absolute and relative Constant–Murley Scores (aCS and rCS) and the subjective shoulder value (SSV). Clinical neurological assessment of the upper extremity was also performed. Subjective satisfaction with the cosmetic result was assessed on a Likert scale from 0 to 100 (subjective aesthetic value [SAV]). Patients were asked to rate the cosmetic outcome independently of the functional outcome.

### Radiological protocol and measurements

**Figure 6:**
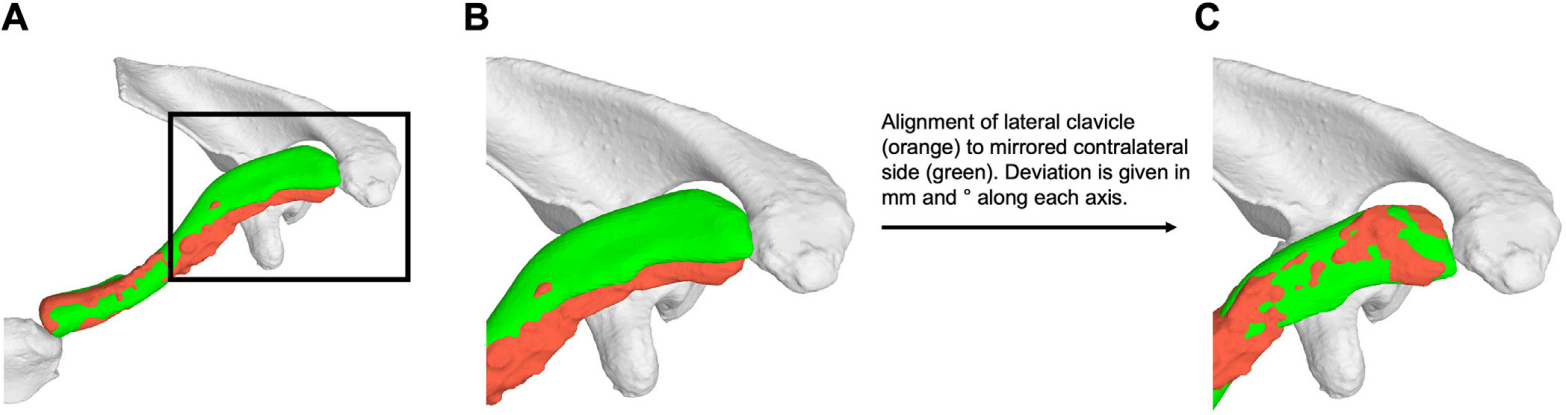
Shows a 3D displacement analysis of a postoperative clavicle (*Left*, orange) compared to the contralateral side (mirrored *Right*, green). (**A**) First, the **medial** third of the postoperative Left clavicle (orange) is aligned to the mirrored contralateral Right side (green). (**B**) Shows the difference in location between the **lateral** third of the postoperative clavicle and the “control”. (**C**) In a next step, the postoperative **lateral** third of the clavicle (orange) is aligned with the mirrored contralateral side (green). The difference between the two positions of the lateral third of the postoperative clavicle (in **B** and **C**) is calculated in mm and ° along each axis and represents the accuracy of the ORIF. *ORIF*, open reduction internal fixation.

**Figure 7:**
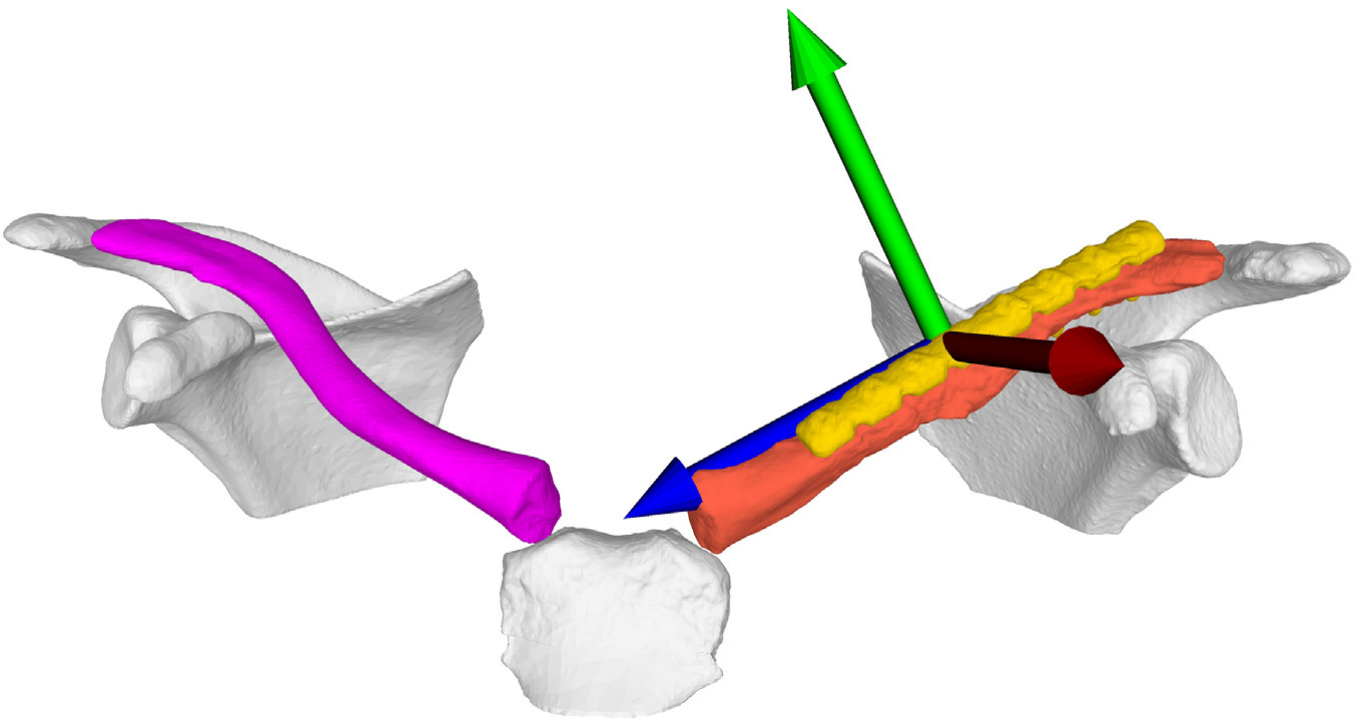
Shows the coordinate system placed according to the International Society for Biomechanics.^26^ The x-axis corresponds to an anteroposterior direction, the y-axis to a craniocaudal direction and the z-axis to a mediolateral direction.

Ultra-low-dose CT^20^ of both clavicles was performed in all patients who did not receive a CT of the clavicle as part of standard follow-up. All CT scans were segmented using a well described standard 3D segmentation software and algorithm (MIMICS version 23, Leuven, Belgium) to generate 3D surface models.^5^^,^^8^ Subsequently, 3D planning software (CASPA, Balgrist CARD, Zurich, Switzerland) was used to analyze these 3D models using a recently published a 3D displacement description method.^27^ This method describes a 3D displacement of an obstacle with only two parameters: a pure shift and a pure rotation. For the pre to postoperative analysis of osteotomies, the method can be used to represent the deviation in height/length and rotation of the lateral bone part of the OT between the planned and the performed surgery.^7^ An analysis using oriented distances and angles in a standard clavicle coordinate system was also performed.^26^ For this purpose, the medial third of the postoperative clavicle was projected onto the contralateral side or planning. The deviations of the lateral third of the postoperative clavicle were then compared to the contralateral side or the planning and analyzed (Fig. 6). The coordinate system was placed according to the International Society for Biomechanics^26^ (Fig. 7). The x-axis corresponds to an anteroposterior direction (positive values indicate an anterior displacement or cranial rotation of the lateral third of the clavicle) the y-axis to a craniocaudal direction (positive values indicate a cranial displacement or posterior rotation of the lateral third of the clavicle) and the z-axis to a mediolateral direction (positive values indicate a medial displacement or posterior rotation around the “main axis” of the clavicle). Finally, a bony union was documented.

### Statistical analysis

All group comparisons were performed with Mann–Whitney–U tests. Variables are reported with a median and range. *P* values below .05 were considered statistically significant. A posthoc sample size calculation showed that for rCS, 87 patients per group, for SSV, 10 patients per group, for 3D transformation shift (TFS), 1129 patients per group, and for 3D transformation angle (TFA), 13 patients per group would be required to detect a statistical difference (with alpha = 0.05 and power 80%). Statistical analysis was conducted with SPSS (version 26.0; IBM Corp., Armonk, NY, USA).

## Results

The groups were comparable in terms of demographic parameters and length of follow-up (median (range); PSI 43 (14-79) months vs. free-hand 50 (12-140) months). Patients in the PSI cohort tended to have undergone more previous surgeries; two-thirds of patients in the PSI group had at least 1 previous surgery whereas less than one-third of patients in the free-hand group had at least 1 previous surgery, although this difference was not significant (median and range: 2 (0-4) vs. 0 (0-6)) (Table I).

**Table I:** Demographic data and group comparison.

	PSI ORIF and/or OT	Free-hand ORIF and/or OT	*P* value†
Total (n)	12	11	
Age at surgery (years)	36 (19; 71)	54 (26; 77)	.124
Sex (m/f)	m:6, f:6	m:8, f:3	
Involved side (l/r)	l:6, r:6	l:9, r:2	
Dominance (l/r)	l:1, r:11	l:4, r:7	
BMI	25 (19, 6; 33, 9)	25.7 (19, 6; 33, 9)	.666
Nicotine (y/n)	y: 4; n: 8	y: 3; n: 8	
Previous surgery (n)	2 (0; 4)	0 (0; 6)	.052
	0 = 4		
	1 = 1	0 = 8	
	2 = 4	1 = 2	
	3 = 1	6 = 1	
	4 = 1		
Indication	Nonunion = 8	Nonunion = 10	
	Malnunion = 2	Refracture of Malnunion = 1	
	Refracture of Malnunion = 2		
ORIF vs. corrective OT	ORIF = 10	ORIF = 11	
	OT = 2		
Augmentation of ORIF or OT	No = 5	No = 4	
	Iliac crest block = 4	Iliac crest block = 2	
	Cancellous bone = 1	Cancellous bone = 2	
	Bone from clavicle resection = 2	Bone from clavicle resection = 2	
		Inductos = 1	
FU (months)	43 (14-79)	50 (12-140)	
Surgery time (min)	115 (28.8)	110 (20)	.7023

Values in median, with range in () or exact values if not applicable.

*PSI*, patient-specific instrumentation; *ORIF*, open reduction internal fixation; *OT*, osteotomy; *BMI*, body mass index; *FU*, follow-up.

† Mann-Whitney *U* Test.

### Clinical outcome data and group comparison

In terms of clinical outcome, patients in both groups had comparably good results without reaching statistical significance (Table II). Although the PSI group fared somewhat better in all parameters, none of the differences were statistically significant or reached the MCID for these scores: the median SSV was 97.5% in the PSI group vs. 90% in the free-hand group (*P* = .221); the SAV was 86.4% vs. 75% (*P* = .111); the CSa was 82.3 points vs. 74.9 points (*P* = .369); and the CSr was 86.7 points vs. 81.9 points (*P* = .588). The mean duration of surgery was also comparable at 115 minutes in the PSI cohort and 110 minutes in the free-hand cohort (*P* = .7023). In the free-hand group, two patients had a postoperative neurological complication. One patient had transient, diffuse, but almost complete motor and sensory plexus palsy, which recovered completely within six weeks of surgery. A second patient had persistent neuropathic, radiating, but dermatomal nonspecific pain after six previous surgeries.

**Table II:** Clinical outcome data and group comparison.

Clinical outcome	PSI ORIF/OT	Free-hand ORIF/OT	*P* value∗
Total (n)	12	11	
SAV (%)	86.4 (10.7)	75 (18.7)	.111
SSV (%)	97.5 (70; 100)	90 (0; 100)	.221
CSa (pts)^+^	82.3 (10.3)	74.9 (26)	.369
CSr (pts)^+^	86.7 (11.3)	81.9 (28.1)	.588
Plexus lesion (n)	0	2	

Values in median, with range in () or exact values if not applicable, significant *P* values are bold.

*PSI*, patient-specific instrumentation; *ORIF*, open reduction internal fixation; *OT*, osteotomy; *SAV*, subjective aesthetic outcome; *SSV*, subjective shoulder value; *CSa*, absolut Constant-Murley Score; *CSr*, relative Constant-Murley Score.

∗ Mann Whitney U Test for non-normally distributed data, t-test for normally distributed data.

### Radiographic outcome data and group comparison

A complete bony union of the nonunion, fracture, or OT was achieved in all cases. The deviations of shift and rotation along the x-, y- and z-axes for the PSI and free-hand cohorts compared to the contralateral native side are shown in Table III.

• Comparison of PSI to contralateral anatomy: the lateral third of the surgically treated clavicle was located on average 4.9 mm too far anteriorly, 2 mm too far inferiorly, and 3.1 mm too short. The average total deviation from the contralateral side was 9.2 mm (3D TFS. Regarding rotation, the lateral third of the clavicle was on average 1.1° superiorly rotated around the anteroposterior x-axis, 0.7° anteriorly rotated around the craniocaudal y-axis and 12° posteriorly rotated around the mediolateral “main” z-axis. The average total error of rotation was 17.7° (3D transformation angle (3D TFA)).
• Comparison of free-hand to contralateral anatomy: the lateral third of the surgically treated clavicle was located on average 1.3 mm too far anteriorly, 1.6 mm too far superiorly, and 2.8 mm too short. The average total deviation from the contralateral side was 9.3 mm (3D TFS). Regarding rotation, the lateral third of the clavicle was on average 1.5° superiorly rotated around the anteroposterior x-axis, 2.7° anteriorly rotated around the craniocaudal y-axis, and 15.1° posteriorly rotated around the mediolateral “main” z-axis. The average total error of rotation was 17.4° (3D TFA).
• Comparison of PSI to planning: the lateral third of the surgically treated clavicle was located on average 2 mm too far anteriorly, 4.1 mm too far inferiorly, and 2.3 mm too short. The average total deviation from planning was 6 mm (3D TFS). Regarding rotation, the lateral third of the calvicle was on average 1.7° superiorly rotated around the anteroposterior x-axis, 0.7° posteriorly rotated around the craniocaudal y-axis, and 3.8° posteriorly rotated around the mediolateral “main” z-axis. The average total error of rotation was 10.8° (3D TFA).

**Table III:** Radiographic outcome data and group comparison.

Radiographic outcome	PSI ORIF/OT	Free-hand ORIF/OT	*P* value†
**Total (n)**	11	6	
**Union (%)**	100	100	
**Radiological comparison to contralateral side**			
**Shift x-axis** (+anterior, −posterior shift)	4.9 (−14.8; 14.4)	1.3 (−7.2; 15.5)	.763
**Shift y-axis** (+superior, −inferior shift)	−2 (−14.5; 1)	1.6 (−10.7; 9)	.315
**Shift z-axis** (+shortening, −lengthening)	3.1 (−6.3; 22.2)	2.8 (−4.2; 4.5)	.763
**3D TFS** (combined shift)	9.2 (2.5; 22.7)	9.3 (5.1; 18.1)	.920
**Rotation x-axis** (anteroposterior axis, +superior)	1.1 (−5.5; 8.3)	1.5 (−8.5; 22.2)	.763
**Rotation y-axis** (craniocaudal axis, +anterior)	0.7 (−15.4; 14.2)	2.7 (−23.8; 7.4)	.841
**Rotation z-axis** (mediolateral axis, +anterior)	−12 (22.3; 10.4)	−15.1 (−23.4; 7.4)	.688
**3D TFA** (combined rotation)	17.7 (10.8; 22.8)	17.4 (11.6; 42.4)	.546
**Radiological comparison to planning**			
**Shift x-axis** (+anterior, −posterior shift)	2 (−5.5; 6.3)	1.3 (−7.2; 15.5)	.239∗
**Shift y-axis** (+superior, −inferior shift)	−4.1 (−6.7; 0.7)	1.6 (−10.7; 9)	.790∗
**Shift z-axis** (+shortening, −lengthening)	2.3 (−0.1; 17.2)	2.8 (−4.2; 4.5)	.970∗
**3D TFS** (combined shift)	6 (3.4; 18.3)	9.3 (5.1; 18.1)	.342∗
**Rotation x-axis** (anteroposterior axis, +superior)	1.7 (−2.4; 7.1)	1.5 (−8.5; 22.2)	.676∗
**Rotation y-axis** (craniocaudal axis, +anterior)	−0.6 (−8.5; 5)	2.7 (−23.8; 7.4)	.569∗
**Rotation z-axis** (mediolateral axis, +anterior)	−3.8 (−23.3; 9.1)	−15.1 (−23.4; 7.4)	.138∗
**3D TFA** (combined rotation)	10.8 (3.1; 23.8)	17.4 (11.6; 42.4)	**.020**∗

For the radiological analysis, the medial third of the postoperative clavicle was aligned with the medial third of the contralateral side. The deviations of the lateral third of the postoperative clavicle compared to the lateral third of the mirrored contralateral side are given as shift and rotation.

Shift in mm and rotation in °.

Values in median, with range in () or exact values if not applicable, significant *P* values are bold.

*PSI*, patient-specific instrumentation; *ORIF*, open reduction internal fixation; *OT*, osteotomy; *TFS*, transformation shift; *TFA*, transformation angle.

∗ Comparison of differences in shift and rotation between PSI/planning and free-hand/contralateral.

† Mann Whitney U Test for non-normally distributed data, t-test for normally distributed data.

In the PSI cohort, the 3D TFA deviation was significantly smaller (PSI/planned vs. free-hand/contralateral: 10.8° (3.1; 23.8) vs. 17.4° (11.6; 42.4); *P* = .020)). There were no other significant differences.

## Discussion

The main finding of the study is that although computer-assisted planning and PSI allow for a more accurate implementation of preoperative planning; it does, however, not document better restoration of anatomy, better healing, or a better clinical outcome. The use of computer-assisted planning and 3D-printed PSI for clavicle non- and malunions has only been reported twice, with three^18^ and four^2^ cases, and has so far not been compared with the conventional technique.

Our first hypothesis that patients treated with PSI would have a significantly better clinical outcome had to be rejected, albeit in a small series of patients. A posthoc sample size calculation showed that 87 patients per group would be necessary to prove a statistical difference for CSr (with a α = 0.05 and a power of 80%), so that the study is clearly underpowered, but it also documents that the differences are so small that very large cohorts are necessary to find a statistical significance. The clinical outcome of patients in both cohorts was very good, both subjectively (SSV and SAV) and objectively (CSa and CSr). Surgical treatment of clavicle nonunion and malunion is associated with higher complication rates and less satisfactory clinical outcomes compared with primary surgical treatment^3^^,^^10^^,^^14^^,^^17^^,^^19^^,^^21^ and remains therefore challenging. This is especially true for nonunion after surgical treatment. Nevertheless, some surprisingly good clinical and radiological results were published more than 20 years ago. Ballmer et al reported 86% very satisfied patients and a 95% union rate 8 years postoperatively.^1^ In our study, two-thirds of patients in the PSI cohort had previous surgery, with an average of two previous operations, and in one-third of patients, a structural iliac crest bone graft was used. In the free-hand group, only about a quarter of the patients had previous surgery, and a structural graft was used less often. The two groups are therefore not fully comparable. More complex situations might have been planned with computer assistance and performed with PSI. The clinical outcome was satisfactory and comparable in both cohorts. One advantage of computer-assisted planning may be that the extent of shortening and contact surfaces can be determined preoperatively, and a decision can be made as to whether structural bone grafting will be required. This in turn allows for better preparation by the surgeon and his team, as well as more accurate information to the patient about the need for ie, iliac crest harvesting.

The second hypothesis, that PSI would result in a more accurate restoration of anatomy, was also rejected. However, when comparing the differences between PSI vs. planning to free-hand vs. the contralateral side, rotation was significantly better restored in the PSI cohort.

To the best of our knowledge, this is the first study to evaluate the radiological outcome using CT and to verify the restoration of the anatomy (in relation to the contralateral side) or the implementation of the preoperative planning on a 3D model. In the literature, only the nonunion rate is usually evaluated, and the length of the clavicle is measured on x-ray or CT.^3^^,^^10^^,^^14^^,^^17^^,^^19^^,^^21^ As the clavicle length on radiographs is projection-dependent,^6^ this information is not reliable. The creation of a postoperative 3D model allows the evaluation of anteroposterior, craniocaudal, and mediolateral displacement as well as rotation compared in relation to the contralateral side or preoperative planning.

The mean 3D shift compared to the contralateral side was 9.2° (PSI) and 9.3° (free-hand). The 3D shift in the PSI cohort consists of the lateral fragment being located more caudally and medially. This can be explained by the application of the reduction guide from cranially. If less soft tissue is removed laterally along the cranial clavicle than medially, the lateral fragment will be pushed down with the guide in good contact. The medial displacement corresponds to a certain amount of shortening, which is to be expected in a nonunion, especially if no bone graft has been inserted after appropriate preparation of the bone ends. The 3D shift in the free-hand cohort is also mainly composed of a medialization of the lateral fragment.

The mean 3D rotation with respect to the contralateral side was 17.7° (PSI) and 17.4° (free-hand). The main component of 3D rotation was due to increased anterior rotation of the lateral fragment (around the mediolateral z-axis).

In summary, the free-hand technique gives better results than expected, so that the bar is high for improvement with computer-assisted planning and PSI. The literature reports a nonunion rate of up to 13% regarding the treatment of clavicle nonunions.^25^ In our two cohorts, union was achieved in all cases. With current technology, the overall anatomy of the contralateral side could only be restored as well as with the free-hand technique. With PSI, the postoperative values were off the planned values by 6 mm 3D TFS (displacement) and by 10.8° 3D TFA (rotation), leaving thus room for improvement. Greater deviations from the planned result could, however, not be correlated with a poorer clinical outcome. At our institution, the planning for an average complex case costs 1650 USD and the printing of the guides 650 USD. Including value-added tax, the costs therefore amount to 2500-2700 USD per case. Whether the extra cost and time involved in PSI are justified for routine cases is something that this paper seriously questions.

### Limitations

The present study has several limitations. First, the retrospective nature of the study makes it impossible to control for all variables. Second, the number of cases is relatively small. A post hoc analysis, however, showed that a very large number of cases would be necessary to document an at least a statistically significant difference. As nonunion or malunion of midshaft clavicle fractures requiring surgical treatment is an uncommon entity, the necessary data would be difficult to collect, and it would have to be questioned whether everybody performing these operations would be in a position to benefit from PSI.

## Conclusions

Surgical treatment of non and malunions of the clavicle was associated with very good clinical results and a 100% union rate. This study, albeit in a relatively small cohort with a follow-up of 4 years, could not document any clinically relevant advantage of 3D planning and personalized operative templating over conventional radiographic planning and free-hand surgical fixation performed by experienced surgeons.

## Disclaimers

Funding: No funding was disclosed by the authors.

Conflicts of interest: The authors or their immediate family, or any research foundation with which they are affiliated did not receive any financial payments or other benefits from any commercial entity related to the subject of this article.
